# User participation and shared decision-making in adolescent mental healthcare: a qualitative study of healthcare professionals’ perspectives

**DOI:** 10.1186/s13034-020-0310-3

**Published:** 2020-01-18

**Authors:** Stig Bjønness, Petter Viksveen, Jan Olav Johannessen, Marianne Storm

**Affiliations:** 1 Centre for Resilience in Healthcare (SHARE), Department for Quality and Health Technology, Faculty of Health Sciences, University of Stavanger, Stavanger, Norway; 2 Department of Psychiatry, Stavanger University Hospital, Stavanger, Norway; 3 Department for Public Health, Faculty of Health Sciences, University of Stavanger, Stavanger, Norway

**Keywords:** Mental health, Psychiatry, Adolescents, Youth, User participation, Shared decision-making, User involvement, Person-centered care, Inpatient

## Abstract

**Background:**

Most mental health problems occur in adolescence. There is increasing recognition of user participation and shared decision-making in adolescents’ mental healthcare. However, research in this field of clinical practice is still sparse. The objective of this study was to explore healthcare professionals’ perspectives on user participation, and opportunities for shared decision-making in Child and Adolescent Mental Health Service (CAMHS) inpatient units.

**Methods:**

Healthcare professionals at CAMHS inpatient units participated in three focus group interviews. Fifteen participants with experience with user participation and shared decision-making were recruited from five hospitals in Norway.

**Results:**

Five themes emerged: (1) involvement before admission; (2) sufficient time to feel safe; (3) individualized therapy; (4) access to meetings where decisions are made; and (5) changing professionals’ attitudes and practices.

**Conclusion:**

User participation and shared decision-making require changes in workplace culture, and routines that allow for individualized mental health services that are adapted to adolescents’ needs. This calls for a flexible approach that challenges clinical pathways and short-stay hospital policies. The results of this study may inform further work on strengthening user participation and the implementation of shared decision-making.

*Trial registration* Norwegian Regional Committees for Medical and Health Research Ethics, reference number 2017/1195

## Background

Most mental health disorders start in adolescence [1, 2]. Adolescence is a unique transitional life period with significant biological, physical, psychological and social changes. These changes increase adolescents’ susceptibility to mental illness. However, low help-seeking behaviors are common among adolescents, and treatment dropout rates are high [3, 4]. Mental illness and the use of mental health services are associated with stigma, and adolescents have a distinct need for autonomy [5–7]. For these reasons, healthcare services need to be adapted to each adolescent’s mental health needs [4, 8]. The prevalence of mental illness among adolescents has forced providers of mental health services to focus on the most acutely ill [9]. Inpatient units are the most common acute mental health service, but research in adolescent inpatient settings is limited [10]. Inpatient admission provides specialized care for adolescents with severe mental health problems and who are in crisis, emphasizing crisis stabilization, assessment and discharge planning [11]. National legislation and policies provide the framework for adolescents’ right to have a say in their healthcare decisions. The United Nations and the World Health Organization (WHO) have called for adolescents with mental health problems to be included in their treatment decisions [12, 13].

User participation in an inpatient context addresses patients’ involvement in their treatment and care, and represents a continuum from being recipients of information to active participants in decision-making [14]. In shared decision-making, healthcare professionals and patients in partnership reach care and treatment decisions, incorporating both evidence-based practices and the patients’ preferences and values [15–17]. User participation and shared decision-making in mental healthcare for adolescents addresses both ethical and legal issues in adolescents’ therapy [17]. Shared decision-making is an interactive process that emphasizes the individual’s values and promotes self-management, in line with the basic principles of person-centered care. Person-centered care is a holistic approach to a health system with respect for the individual person’s abilities, preferences and goals [18–20]. It can be considered as a context for applying user participation and shared decision-making [14], and provides a conceptual framework for this study.

Research suggests shared decision-making contributes to improving self-efficacy, self-esteem, treatment engagement, outcomes and satisfaction [17, 19, 21, 22]. Furthermore, earlier research showed that adolescents wanted to exchange information with clinicians and they wanted to have the autonomy to choose between treatments whenever possible [23]. However, most research on shared decision-making has been conducted within adult mental health settings [3, 24, 25]. Research on shared decision-making for adolescents has mainly focused on whether they have the capacity to participate in treatment decisions, and several studies indicate that they do [16, 17, 19, 26]. Numerous authors argue for increased use of patient participation and shared decision-making to improve the quality of mental healthcare for adolescents [3, 16, 17, 25]. A need to understand inter-professional roles when implementing shared decision-making has been identified [18, 27]. A scoping review identified several approaches to promote and support shared decision-making in child and adolescent mental health, most commonly with the use of decision aids. However, facilitation of shared decision-making also depended on clinicians’ flexibility, effort to mobilize and engage youth, and to develop trust between clinicians and youth [24]. Treatment of mental health disorders among adolescents and the promotion of shared decision-making is complex and there is still limited research in this field [18, 19, 24]. There is a particular need for research exploring professionals’ ability to implement user participation and shared decision-making [17].

The objective of this study was to explore healthcare professionals’ perspectives on user participation and on opportunities for shared decision-making in Child and Adolescent Mental Health Service (CAMHS) inpatient units for adolescents. Our research question was: How can user participation and shared decision-making be facilitated and integrated in CAMHS inpatient units?

## Method

### Focus group interviews

A qualitative, exploratory study design using focus group interviews was carried out to describe healthcare professionals’ perspectives on user participation and shared decision-making within the context of Norwegian CAMHS inpatient settings for adolescents. A focus-group design provides opportunities for social interaction among the participants, and can give the researcher access to multiple perspectives on complex themes [28]. The study was developed with the assistance of two youth co-researchers who had experienced CAMHS services. Youth involvement in research strengthens the relevance for youth as it includes their perspectives. Involvement should take place at conceptualization of and throughout a research project, in order to avoid piecemeal involvement [29]. The co-researchers participated in designing the project plan, the information sheets for focus group participants and the interview guide. They received information about and provided input to the study at meetings throughout the study.

### Study setting

The study participants were healthcare professionals working in Norwegian CAMHS inpatient settings for adolescents aged 13–18 years. The focus group interviews took place with employees from CAMHS units that had been engaged in a quality improvement project with *The Change Factory* from 2016 to 2018. *The Change Factory* is a non-profit service user interest organization. As part of their philosophy, they advocate the right of youth who have used mental health services to participate in developing and reviewing the services. They consider hearing youths’ voices to be a key part of the process to improve and ensure the provision of high quality mental health services for those who receive them [30].

### Recruitment and participants

The focus group participants were recruited from five CAMHS that were part of the quality improvement project set up by *The Change Factory*. Recruitment took place in parallel with national events organized by *The Change Factory*. We recruited a purposive sample of experienced healthcare professionals with both therapist and management perspectives on user participation and shared decision-making. Three focus group interviews were carried out between June and November 2018, including a total of 15 participants (eight women and seven men). All participants had clinical work experience within CAMHS. Five participants who worked as clinicians were interviewed in a separate focus group so that they could speak candidly without the presence of their managers. Although the remaining ten participants also had a background as clinicians and all (except one) were still in clinical practice, they also served in management positions. These ten participants were interviewed in two focus groups with four and six participants respectively. Participants’ professional background and work experiences are provided in Table 1. Their age and gender have been omitted to prevent identification.

**Table 1:** Study participants’ professional background and work experience

Participant ID number	Focus group number	Professional background/education	Work experience in CAMHS
1	1	Social educator	2 years
2	1	Social educator	10 years
3	1	Child welfare officer	4 years
4	1	Social worker	15 years
5	1	Psychologist	35 years
6	1	Social educator	17 years
7	2	Psychiatrists	22 years
8	2	Psychologist	10 years
9	2	Social educator	25 years
10	2	Psychiatric nurse	24 years
11	3	Family therapist	11 years
12	3	Doctor	9 months
13	3	Social worker	17 years
14	3	Social worker	13 years
15	3	Family therapist	8 years

### Data collection

The three focus group interviews were organized as 90-min conversations led by the first author (SB). Interviews were audio-recorded and the second author (PV) took notes to facilitate understanding of the recording. One focus group took place in parallel with a meeting jointly organized by *The Change Factory* and the CAMHS. The two other focus groups took place in two CAMHS units in two health regions. An interview guide developed in collaboration with youth co-researchers was used. The content was informed by published literature [9, 19, 24, 31]. The interview questions were open-ended, such as: *What do you think about adolescents being involved in treatment decisions?* and *How does your workplace facilitate user participation?* The participants were encouraged to share their experiences and give examples. Questions were developed to explore participants’ perspectives on user participation and opportunities for shared decision-making in Child and Adolescent Mental Health Service (CAMHS) inpatient units for adolescents. The sample size and information power were discussed by two researchers between interviews (SB, MS). According to Malterud, Siersma, and Guassora [32] the information power is strengthened through the use of a narrow research aim, and thematically relevant dialogue with significant interaction among focus group participants. Rich data add depth to gain insight and understanding, and sufficient rich data enabled the researchers to see when information was repeated [33, 34]. Following the three interviews, the data was considered rich, containing information of high relevance to the study objectives.

### Data analysis

Audio-recorded focus group interviews were transcribed and analyzed using systematic text condensation, as described by Malterud [32]. Two researchers (SB, MS) read all interview transcripts independently to identify preliminary themes of relevance to the research question. Seven preliminary themes were identified. The two researchers (SB, MS) then discussed and reached agreement on five code groups based on preliminary themes, and then identified units of meaning related to the code groups. At that point, units of meaning were organized into subgroups and the contents in each subgroup were condensed. The second author (PV) was involved in the third analysis stage, and consensus was reached among the researchers. In the final stage, the contents of the condensates were synthesized to present summarized descriptions of healthcare professionals’ perspectives on how user participation and shared decision-making can be facilitated and integrated. Table 2 shows an example of the analysis process.

**Table 2:** Example of the analysis process

Preliminary themes	Code group	Subgroups	Condensate
Meetings Framework Initiatives Routines	Access to meetings where decisions are made	1. Change work culture 2. Implementing routines	Excerpt from the subgroup 2 implementing routines: Participation requires changes in routines and meeting structure. Previously we made decisions in pre-meetings, and then we gathered all the professionals and called in the adolescents. There were too many people present at the meetings

## Results

The five code groups that emerged from the analysis represent five major themes: (1) involvement before admission; (2) sufficient time to feel safe; (3) individualized therapy; (4) access to meetings where decisions are made; and (5) changing professionals’ attitudes and practices. All of these themes describe factors healthcare professionals perceived as necessary to facilitate user participation and shared decision-making in CAMHS inpatient units for adolescents.

### Involvement before admission

The interview participants emphasized the importance of adolescents’ experience of being at the center of the services. Admission to an inpatient unit under parental pressure or threats of involving child welfare services were examples not being at the center. Adolescents’ voices and views were not always clearly presented in referral letters. The participants emphasized the importance of providing adolescents with sufficient information about treatment and user participation prior to admission to the inpatient unit. Without such information, adolescents would be unable to make an informed decision about participating in treatment and being involved in decisions concerning their treatment course. According to the participants, healthcare professionals should establish a dialogue with the adolescents prior to admission and express a wish to cooperate with and to involve them in shared decision-making. Care planning meetings before admission to inpatient care were considered important to clarify adolescents’ expectations and understanding of their views of treatment goals. This was perceived to reduce the use of involuntary treatment. Involvement in the early stages of the treatment course reduced motivational problems in adolescents, which several participants highlighted as important to user participation. One participant described the challenges posed by the absence of dialogue prior to admission:

*What the adolescents themselves want to get out of the treatment has not been discussed before admission. So, when they arrive, they either say nothing or they just partly agree. What can we do if they don’t say anything? We then have a major dilemma with regards to creating their sense of ownership of the stay in our unit.* (Participant 6)

Shared decision-making was described as a combination of professional knowledge, clinical experiences and user knowledge. Without a common understanding of treatment goals, professionals and adolescents work against each other, and it does not matter how good professionals’ intentions or ideas are. Involvement before admission was considered a prerequisite for shared decision-making later on in the treatment. Several participants expressed frustration over situations in which adolescents had expected help without contributing to their planning and treatment. They insisted on the importance of clarifying adolescents’ and clinicians’ expectations at the outset. To receive customized help, adolescents must be willing to tell clinicians what they needed. User participation was not described as facilitating, but as involving and challenging adolescents to become “captain of their own ship.”

*There is an element of expected participation. At that point, I think there has been a change in how we consider user involvement and participation. We have worked quite a lot on turning the mindset over to; “In fact, we actually expect you to join us, to let us know what you need.”* (P1)

### Sufficient time to feel safe

The participants went on to discuss the importance of the time needed in therapy in order for adolescents to feel safe. Establishing a good relationship with adolescents was necessary to make them feel safe enough to participate in treatment decisions. Sufficient time was considered crucial to establish such a relationship. Several participants cited time constraints in their daily work as a significant barrier to the establishment of a safe patient-practitioner relationship. Short-stay hospital policies and procedures such as diagnosing and initiating treatment plans shortly after admission, exacerbate the time pressure. Planning was often done by therapists who rushed to reach treatment goals without taking the time to consult with the adolescents. It was also difficult for adolescents to make important decisions shortly after admission. Adolescents could be vulnerable or defiant and therefore needed a long time before they were willing or able to become participants in decision-making. Some participants attributed this at least partly to adolescents’ experiences with hospital admission, where others had made decisions on adolescents’ behalf.

*I believe [the patient*-*practitioner] relationship is a prerequisite for user participation. This must be in place in order for participation to function. The [patient*-*practitioner] relationship and user participation should be one and the same.* (P15)

According to focus group participants, many adolescents would have liked more time to discuss what they saw as the core of their problem, rather than cooperating just to reach a quick diagnosis and follow a standardized clinical pathway. Some participants discussed time constraints as follows:

*[Each adolescent] is supposed to get in and out as quickly as possible, because “there is always another one who needs to enter.” I wonder if such issues contribute to reduce user participation and shared decision*-*making.* (P14)

*It’s a bit paradoxical. User involvement is about relations and caring for the individual. We are supposed to help them make choices, but we won’t be able to do so rushing towards a goal. (P11)*

### Individualized therapy

Although clinical pathways are designed to ensure user participation, several focus group participants pointed out that they do not take sufficient account of individual adolescents’ needs. Adolescents undergoing psychotherapy often have complex needs, and this complexity makes it difficult to predefine a treatment course and duration.

Nevertheless, the participants described it as possible to reach treatment goals if practitioners did not insist on sticking to standardized practices and regulations. Some professionals said that going beyond therapy recommendations challenged their sense of professionalism; others thought this strengthened it. Many participants emphasized the need for individual approaches for adolescents who had the most severe mental health problems, although it sometimes meant going beyond clinical pathways. Those adolescents were also considered to have the greatest need for user participation. Two participants talked about the importance of flexibility:

*We are supposed to work efficiently and reduce costs through short, targeted hospital admissions […] It’s not easy to get an adolescent with huge challenges and secrets to follow a recipe within a specific time frame.* (P13)

*Then one must dare to not always play by the book in terms of clinical pathways. It is all about flexibility, I think […] For example, some adolescents need longer admission. I had one adolescent not very long ago where we made an exception because it was necessary and reassuring for her and her family. It may actually turn out to be timesaving.* (P12)

However, because of resource constraints, not all adolescents in an inpatient unit could be offered the same options. One participant referred to it as “different types of shared decision-making” (P7), suggesting that, due to financial constraints, the same “menu” of choices could not be offered every adolescent.

### Access to meetings where decisions are made

A central issue was how best to hear the individual adolescent’s voices. Important factors included which forums they should attend, workplace culture and staff attitudes. Meetings were described as important decision-making arenas and the participants considered it “best practice” to invite adolescents to formal treatment meetings. In the past, therapists made decisions without consulting the adolescents. A culture of “no decision without participation” had been introduced. Informal meetings among professionals without adolescents’ participation could affect the basis of decision-making. Such a practice was not considered to be part of a culture that contributed to user participation. Cooperation with adolescents who have had experience with the services was important in raising awareness among healthcare professionals and leaders.

*Something happened with the attitudes after we started to cooperate with The Change Factory. It has become natural for both healthcare personnel and us leaders to bring adolescents into team*-*meetings. There is no question whether they should participate. In my experience, it is a mindset: Yes, of course, they should be involved. If not, we or they must give reasons for why they should not join.* (P6)

There was a consensus among the focus group participants that adolescents needed a role in important decisions and that meetings should be open to them. The planning and structure of meetings had in the past not been conducive to adolescents’ participation. For example, the meetings had too many attendees, which had been a deterrent to adolescents speaking up or even coming. The focus group participants emphasized practices such as planning meetings with the adolescents well in advance, to agree on meeting agendas and to ask if there was anyone that the adolescent would like to invite to the meetings. Some focus group participants acknowledged that they were still discussing within their unit whether adolescents should participate during entire meetings. They needed more time to adapt before they could fully embrace user participation. Others had established routines for participation in meetings, and some used simulation techniques with representatives from *The Change Factory* in order to improve practitioners’ user-participation skills.

*The biggest change has been to make the treatment meetings available to adolescents. We thought: How can we make it safe enough? The treatment meetings have been too large, too many people attending. Therefore, adolescents simply did not attend their own meetings. It is perhaps the biggest and most important change in recent times.* (P4)

### Changing professionals’ attitudes and practices

Some focus group participants viewed user participation as a paradigm shift in clinical work. They discussed how increased user participation required them as professionals to meet adolescents with greater warmth and care. Several referred to the youth from *The Change Factory*, who expressed a need for “love” within the context of the patient-practitioner relationship. Healthcare professionals thought that this challenged their professional identity and even took away some of their responsibility for the treatment. Some pointed out that they had been trained to take responsibility for their professional decisions, and that their role as a therapist was challenged, as they have never had to learn how to manage shared decision-making. However, other focus group participants claimed that this was a misunderstanding. They perceived user participation as a means to reinforce professionalism because including adolescents in decision-making processes required them to be updated and confident about their professional role. Some professionals who held manager positions said it was difficult to change the attitudes of clinical staff towards user participation. They described this as an ordeal that they would prefer to avoid. Nevertheless, participants agreed that without a “caring approach,” adolescents were likely to be less engaged in therapy or might even reject therapy altogether.

*It is a bit challenging for us therapists who suddenly get a dual role in being caregivers in addition to therapists. In other words, adolescents require a caring therapist. And we have been taught no, don’t mix those roles.* (P7)

A high degree of user participation was considered easiest to achieve when adolescents already had a family and a social network that provided them with the necessary care. Parental support facilitated shared decision-making, as opposed to those adolescents who lacked such support or had conflicts within their family. Healthcare professionals could not collaborate on treatment decisions with adolescents if they did not support the adolescents’ perspectives. To gain the trust of these adolescents, the professionals had to accept that some adolescents had rejected their parents. Many participants were concerned about what might happen when parents were no longer part of their adolescents’ treatment. This resulted in a sense that they were working in “the space” between healthcare and child welfare services. When adolescents’ care and basic needs were not covered, shared decision-making became difficult because adolescents had nowhere to go and treatment had to be postponed.

*It is not always clear when they are referred here, but then we discover that there is no home*-*care base. What will user involvement become then? They experience powerlessness in their lives beyond anything imaginable.* (P11)

## Discussion

This exploration of healthcare professionals’ perspectives has implications for service design and service delivery in the field of CAMHS, by providing insights into potential means to increase user participation and opportunities for shared decision-making. It requires routines for involving adolescents in advance of and throughout inpatient treatment and individualized mental health services. Training for healthcare professionals, changes in workplace culture and flexible approaches adapted to adolescents’ particular needs are recommended.

### No decision without participation

The findings are in accordance with policies that place patients’ needs, wishes and preferences at the center of clinical decisions, which is also in line with core principles of person-centered treatment. Care planning without adolescents’ participation can lead to disengagement from the therapeutic process [35]. Coulter and Collins [15] suggest that shared decision-making is the norm to ensure patients’ perspectives and participation are heard and integrated into treatment decisions. To ensure that this becomes a reality, arenas for shared decision-making must be identified. This study points out two important decision arenas: (1) care planning meetings with adolescents where goals are set and the decision about admission to the inpatient unit is made; and (2) adolescents’ participation in meetings during treatment.

Several studies have stressed the importance of identifying goals and encouraging participation in decision-making early in the treatment process [17, 36, 37]. Giving adolescents an early experience of being true partners in decisions may support their autonomy and create a sense of agency within treatment [38]. The participants in our study suggest that involving adolescents even before hospital admission will improve user participation throughout the treatment course. However, this can present practical challenges in acute treatment. Interdisciplinary collaboration between primary health services and outpatient clinics, as well as outreach services prior to admission, provide opportunities for care planning with the adolescent before admission to inpatient care. The focus group interviews also suggest that such practice can contribute to the reduction of involuntary treatment.

User participation and shared decision-making are linked to treatment plans and meetings [36, 39]. Participants in this study viewed closed-door meetings or being asked to join the meetings after professionals have already made the decisions as barriers to shared decision-making. Making meetings accessible to adolescents is crucial for shared decision-making. This implies rescheduling meetings to accommodate adolescents’ needs, and is consistent with previous research [39, 40].

### The patient-practitioner relationship and individualized treatment

A trustful patient-practitioner relationship is one of the most robust predictors for treatment satisfaction and outcome [25, 41, 42]. Similar to a study by Oruche et al. [43], this study notes that treating adolescents as partners is necessary to improve their participation in decision-making. However, healthcare professionals are obligated to conduct clinical assessment, diagnose and initiate treatment within certain time limits, which can be referred to as clinical pathways. Clinical pathways are interventions to improve the quality of healthcare, and they detail the steps in a course of treatment [44]. They have proven most effective when the course of treatment is predictable [45]. According to the participants, clinical pathways have the potential to ensure user participation and evidence-based practice. However, psychotherapy with adolescents is often unpredictable and our results indicate a dilemma between clinical pathways, hospitals’ short stay-policies and user participation. It is also necessary to recognize the time needed to overcome adolescents’ possible skepticism towards inpatient care and individually adapt participation in decision-making [4, 42]. Shared decision-making can be conceptualized on a continuum between clinician-led decisions and decisions left entirely to the patient [18, 31]. Hayes et al. [10] argue that adherence to rigid procedures can result in adolescents not receiving individualized treatment. Our study emphasizes the need for a flexible approach by healthcare professionals, including an individual assessment of each adolescent. Flexibility and individualized treatment are well-known facilitators for implementing shared decision-making [19, 21, 37, 46].

Participation and shared decision-making were described as particularly challenging in the treatment of adolescents with severe illness, those who come from disadvantaged backgrounds or who need child welfare services. Nevertheless, it is crucial to develop services not only adapted to highly functioning adolescents [47]. Furthermore, it was considered even more important to maintain a person-centered approach to care; healthcare professionals must offer warmth and care to enhance user participation. Findings suggest that clinicians must assess the alternatives that are available to each adolescent, in order to achieve treatment goals. Shared decision-making may be applied in different ways and to different degrees. For example, adolescents with complex or severe mental problems may need more time and/or a greater range of choices.

### Setting the stage for user participation and shared decision-making

Studies indicate a need for a culture change to develop user participation and to support shared decision-making in mental healthcare [20, 31]. Our results indicate that user participation in CAMHS inpatient units is related to the therapist’s interest in caring for and engaging in the adolescent’s situation, and that there is a need to improve staff training and ward culture to manage user participation and shared decision-making processes. Placing adolescents in control over the decisions that affect them has traditionally not been a part of healthcare professionals’ training. Considerable efforts are required, if the changes to the work culture can be expected [31, 48, 49]. This includes leadership encouragement, organizational support and communication of the benefits of user participation and shared decision-making to clinicians [47]. To facilitate opportunities for shared decision-making, we recommend that the work culture should gradually incorporate a person-centered approach to care. Collaboration with experienced young service users can therefore contribute to awareness and willingness to establish routines for “no decision without participation”. Experienced service users challenge existing assumptions about adolescents’ needs, and it is important to involve them in quality improvement initiatives [50, 51].

### Limitations

We conducted three focus group interviews with 15 participants. Additional individual interviews could have generated more information. A small sample of participants with experience in Norwegian CAMHS and who had taken part in a quality improvement project may also affect transferability. It should be noted that this study is not an evaluation of *The Change Factory* project but it was considered important to recruit participants with clinical experience with user participation and shared decision-making in mental health services. Shared decision-making can be used to incorporate personal values and evidence-based practice into treatment decisions. This study does not include experiences with shared decision-making tools, but considers the decision-making process in a wider user participation perspective. Within this context we have explored opportunities for shared decision-making in CAMHS inpatient units.

## Conclusion and implications

The results of this study suggest healthcare professionals consider it crucial to implement strategies for training, workplace culture and establishment of routines to increase adolescents’ participation in their care. A common understanding of user participation and treatment goals should be developed before admission to the inpatient unit, and adolescents should be invited to meetings throughout the treatment. A participation model providing opportunities for shared decision-making should be adapted not only to highly functioning adolescents. The study underlines the importance of professionals’ being relationally oriented, emotionally engaged and willing to adapt approaches to individual adolescents’ needs. Hence, clinical pathways should merely be guidelines. We suggest services based on a person-centered approach and a user participation-model as a preferred model dealing with acute mental illness, addressing ethical issues and shaping the future mental healthcare. From a policy perspective, we recommend training in shared decision-making and person-centered treatment to be part of healthcare professionals’ formal education.

The results from this study can inform further work on strengthening user participation and on developing and implementing shared decision-making. Further research should explore adolescents’ experiences with shared decision-making. There is also a need for validated shared decision-making measures and effect-studies for outcomes after shared decision-making in adolescent mental healthcare settings.

## Data Availability

The datasets generated and analyzed in this study are not publicly available because of individual privacy considerations and limitations from ethics approval. Information about data are available from the corresponding author on reasonable request. Note that verbatim-transcribed data are in Norwegian.
